# The primary carbon metabolism in cyanobacteria and its regulation

**DOI:** 10.3389/fpls.2024.1417680

**Published:** 2024-07-05

**Authors:** Stefan Lucius, Martin Hagemann

**Affiliations:** Department Plant Physiology, University of Rostock, Rostock, Germany

**Keywords:** synechocystis, glycolysis, CP12, Pirc, inorganic carbon (Ci)

## Abstract

Cyanobacteria are the only prokaryotes capable of performing oxygenic photosynthesis. Many cyanobacterial strains can live in different trophic modes, ranging from photoautotrophic and heterotrophic to mixotrophic growth. However, the regulatory mechanisms allowing a flexible switch between these lifestyles are poorly understood. As anabolic fixation of CO_2_ in the Calvin-Benson-Bassham (CBB) cycle and catabolic sugar-degradation pathways share intermediates and enzymatic capacity, a tight regulatory network is required to enable simultaneous opposed metabolic fluxes. The Entner-Doudoroff (ED) pathway was recently predicted as one glycolytic route, which cooperates with other pathways in glycogen breakdown. Despite low carbon flux through the ED pathway, metabolite analyses of mutants deficient in the ED pathway revealed a distinct phenotype pointing at a strong regulatory impact of this route. The small Cp12 protein downregulates the CBB cycle in darkness by inhibiting phosphoribulokinase and glyceraldehyde 3-phosphate dehydrogenase. New results of metabolomic and redox level analyses on strains with Cp12 variants extend the known role of Cp12 regulation towards the acclimation to external glucose supply under diurnal conditions as well as to fluctuations in CO_2_ levels in the light. Moreover, carbon and nitrogen metabolism are closely linked to maintain an essential C/N homeostasis. The small protein PirC was shown to be an important regulator of phosphoglycerate mutase, which identified this enzyme as central branching point for carbon allocation from CBB cycle towards lower glycolysis. Altered metabolite levels in the mutant Δ*pirC* during nitrogen starvation experiments confirm this regulatory mechanism. The elucidation of novel mechanisms regulating carbon allocation at crucial metabolic branching points could identify ways for targeted redirection of carbon flow towards desired compounds, and thus help to further establish cyanobacteria as green cell factories for biotechnological applications with concurrent utilization of sunlight and CO_2_.

## 1 Introduction

Cyanobacteria are a phylum of Gram-negative prokaryotes from the domain of bacteria. At least 2.7 billion years ago, they evolved oxygenic photosynthesis, a process in which light energy is used for water splitting to generate chemical energy in the photosynthetic electron transport chain combined with the release of molecular oxygen (O_2_) as a byproduct (e.g., Hohmann-Marriott and Blankenship, 2011; Sánchez-Baracaldo and Cardona, 2020). The long-term evolution of cyanobacteria allowed adaptation to numerous habitats including extreme locations like salt lakes (Alvarenga et al., 2018) or desert soil crusts (Oren et al., 2019). Cyanobacteria are important drivers of the global carbon cycle, with picoplanktonic *Synechococcus* and *Prochlorococcus* strains alone accounting for at least 25% of all marine primary production (Flombaum et al., 2013). Several cyanobacterial strains are also able to fix atmospheric dinitrogen and contribute to the global N cycle (Herrero et al., 2016). The photosynthetic CO_2_ fixation was transferred from ancient cyanobacteria to eukaryotes by endosymbiosis, which essentially initiated the development of plastids in the plant kingdom approximately 1.5 billion years ago (Hedges et al., 2004). Therefore, several cyanobacterial biochemical pathways and physiological traits remained conserved in plants. This makes cyanobacterial metabolism an attractive subject of fundamental research for plant photosynthesis and energy metabolism. Moreover, the natural production and accumulation of biopolymers such as glycogen, cyanophycin, or polyhydroxybutyrate (PHB) (Santos-Merino et al., 2019) and the expression of pathways for feedstock production highlights cyanobacteria as suitable green cell factories for application in biotechnology (Hagemann and Hess, 2018).

### 1.1 The primary carbon metabolism in cyanobacteria

#### 1.1.1 Inorganic carbon-concentrating mechanisms (CCM)

Cyanobacteria evolved the CCM in response to evolutionary pressure caused by the increase of O_2_ and decrease of CO_2_ concentration in their environment that followed the rise of oxygenic photosynthesis (Raven et al., 2017). Furthermore, the solubility of CO_2_ is low in aquatic habitats making CO_2_ less available compared to air. The specific requirement for such a mechanism lies in the biochemical properties of RuBisCO, because it has a low affinity and specificity for CO_2_. Therefore, in addition to the carboxylation reaction adding CO_2_ to ribulose 1,5-bisphosphate (RuBP) to generate two molecules of 3-phosphoglycerate (3PGA), RuBisCO can also incorporate O_2_ into RuBP via its oxygenase activity, resulting in the production of one molecule each of 3PGA and 2-phosphoglycolate (2PG) (Kaplan and Reinhold, 1999). The metabolite 2PG is toxic for oxygenic photosynthetic organisms, because it can inhibit CBB cycle enzymes such as triose-phosphate isomerase and seduheptulose 1,7-bisphosphatase (Anderson, 1971; Flügel et al., 2017). Hence, 2PG needs to be metabolized by the photorespiratory salvage pathway (Eisenhut et al., 2008a).

The CCM reduces the oxygenase reaction of RubisCO to a flux below 1% in cyanobacteria (Huege et al., 2011; Young et al., 2011), because Ci is enriched in the cells. Model strains such as *Synechocystis* sp. PCC 6803 (hereafter *Synechocystis*) possess five well-described Ci uptake systems to accumulate bicarbonate in the cytoplasm: three bicarbonate transporters BCT1 (Omata et al., 1999), SbtA (Shibata et al., 2002), and BicA (Price et al., 2004), which are located in the plasma membrane, and two complexes NDH-1_3_ and NDH-1_4_ that are thylakoid-bound and convert CO_2_ to HCO_3_^-^ (Rae et al., 2013). Bicarbonate then diffuses into cyanobacterial micro-compartments called carboxysomes, which harbour the enzymes carbonic anhydrase (CA) and RuBisCO. Here, bicarbonate is converted back to CO_2_ by CA while the carboxysome shell prevents the loss of CO_2_ and thus increases its concentration around RuBisCO (reviewed in Hagemann et al., 2021).

In aquatic habitats, cyanobacteria regularly experience fluctuating levels of Ci in depending on pH, temperature, and salinity that influence the solubility of CO_2_ and conversion to bicarbonate (Kupriyanova et al., 2023). Therefore, regulation of the primary carbon metabolism involves adjusting the CCM as a way to maintain CO_2_ fixation efficiency in limiting conditions. In contrast to the carbon metabolism (see below), the CCM activity is mainly regulated at transcriptional level (Burnap et al., 2015; Hagemann et al., 2021). The transcription factor NdhR represses the expression of genes in the largest Ci regulon in *Synechocystis* under high CO_2_ conditions (Figge et al., 2001; Klähn et al., 2015). NdhR is fine-tuned by 2OG and 2PG to sense intracellular C/N ratios (Muro-Pastor et al., 2001; Jiang et al., 2018). Another transcription factor, CmpR, activates the BCT1 transporter under low Ci conditions (Omata et al., 2001; Nishimura et al., 2008). Moreover, there are indications for the cyanobacterial regulator CyAbrB2 to regulate the repressor NdhR and to affect PSI subunit expression during acclimation to Ci limitation (Ishii and Hihara, 2008; Orf et al., 2016a). In addition to these transcriptional regulations, the cyanobacterial CCM is also regulated by the PII-paralog SbtB, which is co-expressed with the sodium/bicarbonate symporter SbtA in the *sbtAB* operon (Selim et al., 2018). It can bind to adenyl-nucleotides including the second messenger cAMP and c-di-AMP and is involved in the regulation of several aspects of Ci acclimation in *Synechocystis* and other cyanobacteria (reviewed in Mantovani et al., 2023).

#### 1.1.2 Organic carbon uptake

Some cyanobacterial strains including *Synechocystis* can also use external organic carbon as basis for heterotrophic life style (Stebegg et al., 2023). This includes the utilization of glucose that can be imported into the cell via the GlcP transporter (Schmetterer, 1990). The imported glucose is phosphorylated by the hexokinase as it enters the central carbon metabolism. From there, it can be metabolized and oxidized by following glycolytic routes. Fructose can also pass GlcP but is toxic for *Synechocystis* (Joset et al., 1988), however, it can be metabolized by other strains (Stebegg et al., 2023). The ability to take up external glucose is significant for *Synechocystis*, as it enables the cells to grow heterotrophically in conditions where photosynthetic CO_2_ fixation is not feasible like dark phases. Moreover, extracellular glucose can be also used in the light for mixotrophic growth. Previous studies confirmed that CO_2_ fixation in the CBB cycle is not interrupted by supply of external glucose in light (Nakajima et al., 2014). Interestingly, growth rates of *Synechocystis* are found to be higher in mixotrophic conditions than the sum of photoautotrophic and heterotrophic yield (Lopo et al., 2012; Chen et al., 2016), suggesting mixotrophy as a separate lifestyle instead of just a mechanism to overcome nutrient scarcity.

#### 1.1.3 Significance of carbon metabolic routes

**Figure 1:**
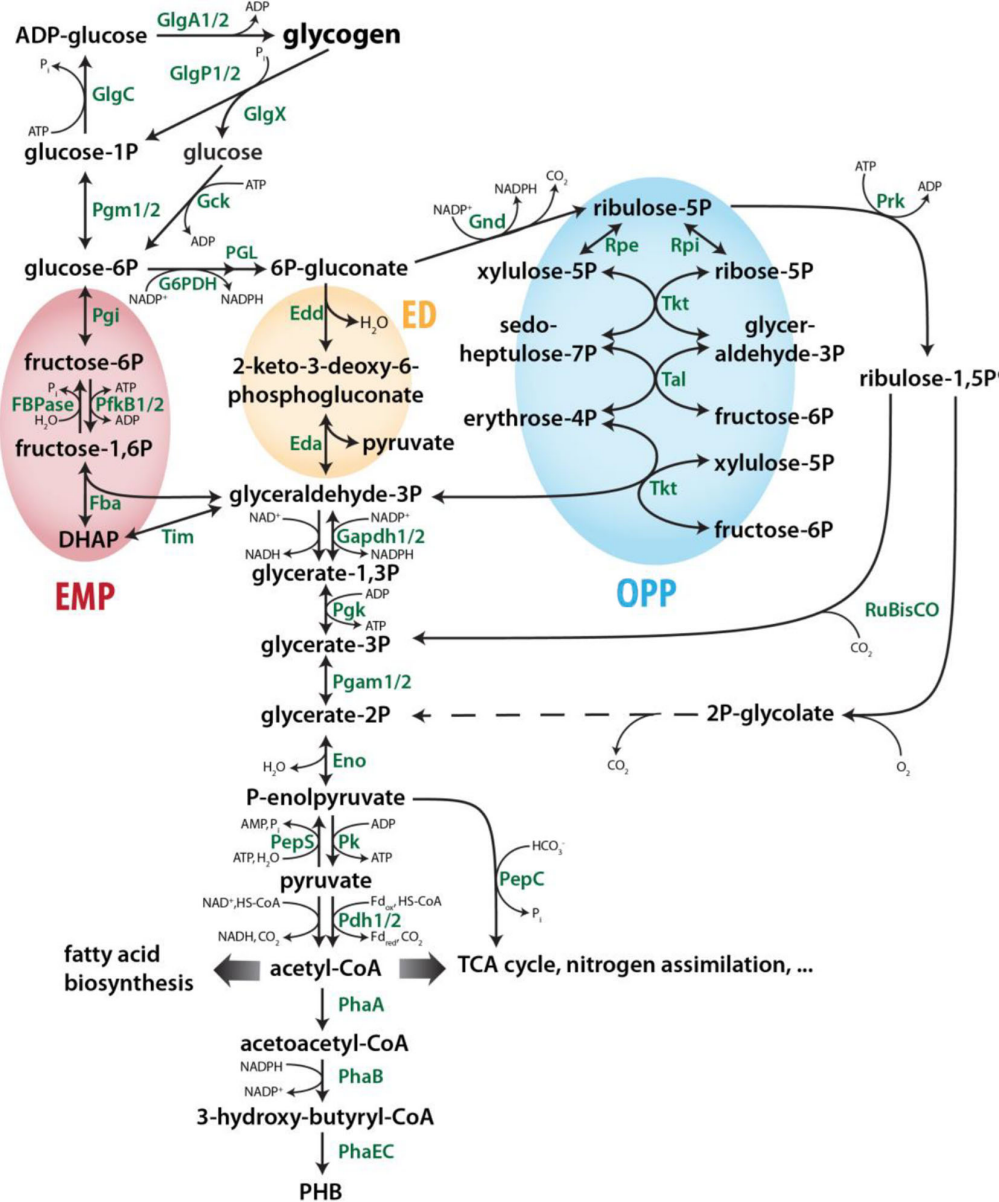
Overview of the central carbon metabolism in cyanobacteria such as *Synechocystis* sp. PCC 6803. Adapted from Koch et al. (2019). Dotted lines represent several enzymatic reactions. The EMP, ED and OPP pathways are highlighted in green, blue and yellow, respectively. Please note that many reactions/enzymes in the interconversion glyceraldehyde-3P and ribulose-5P are shared by the OPP and CBB pathways albeit in opposite directions. Eda, KDPG aldolase; Edd, Phosphogluconate dehydratase; Eno, Enolase; Fba, Fructose bisphosphate aldolase; FBPase, Fructose 1;6-bisphosphatase; G6PDH, Glucose 6-phosphate dehydrogenase; Gapdh, Glyceraldehyde 3-phosphate dehydrogenase; Gck, Hexokinase; GlgA, Glycogen synthase; GlgC, Glucose 1-phosphate adenylyltransferase; GlgP, Glycogen phosphorylase; GlgX, Glycogen debranching enzyme; Gnd, 6-phosphogluconate dehydrogenase; Pdh, Pyruvate lycolgenase; PepC, Phosphoenolpyruvate carboxylase; PepS, Phosphoenolpyruvate synthase; PfkB, Phosphofructokinase; Pgam, Phosphoglycerate mutase; Pgi, Glucose 6-phosphate isomerase; Pgk, Phosphoglycerate kinase; PGL, 6-Phosphogluconolactonase; Pgm, Phosphoglucomutase; PhaA, Acetyl-CoA acetyltransferase; PhaB, Acetoacetyl-CoA reductase; PhaEC, PHB synthase; PHB, Polyhydroxybutyrate; Pk, Pyruvate kinase; Prk, Phosphoribulokinase; Rpe, Ribulose 5-phosphate 3-epimerase; Rpi, Ribose 5-phosphate isomerase; RuBisCO, Ribulose 1;5-bisphoshate carboxylase/oxygenase; Tal, Transaldolase; Tim, Triose-phosphate isomerase; Tkt, Transketolase.

The central carbon metabolism of the model strain *Synechocystis* comprises several biochemical pathways that allow the organism to live photoautotrophically, heterotrophically, and mixotrophically (Pelroy et al., 1972; Mills et al., 2020). These different modes of lifestyle are fundamentally defined by the direction of enzymatic reactions in the respective central metabolic routes. In that way, a complex metabolic network is created to intertwine anabolic reactions of the CBB cycle with gluconeogenesis for Ci reduction and storage, and catabolic reactions of glycolytic pathways that oxidize organic carbon sources like stored glycogen and imported glucose (Xiong et al., 2017). For cyanobacteria like *Synechocystis*, which do not possess organelles or major cellular compartments, a tight regulation of metabolic routes and overlapping central carbon pathways is essential to react and to adjust towards rapid environmental changes like fluctuations of light or CO_2_ availability (Jablonsky et al., 2016). **Figure 1** gives an overview of main routes in the primary cyanobacterial carbon metabolism.

#### 1.1.4 Anabolic carbon routes and glycogen formation

As in all oxygenic phototrophs, the principal anabolic pathway for the reduction of Ci to organic sugars and metabolic intermediates is the CBB cycle among cyanobacteria (Durall and Lindblad, 2015). RuBisCO binds CO_2_ onto RuBP in the carboxysome producing 3PGA. Then, 3PGA is mainly metabolized for the regeneration of the precursor molecule RuBP to conclude the CBB cycle. In this process, the key enzyme glyceraldehyde 3-phosphate dehydrogenase 2 (GapDH2, Sll1342 in *Synechocystis*, Koksharova et al., 1998) requires NADPH from the photosynthetic light reaction to convert glycerate 1,3-bisphosphate (G1,3BP) to glyceraldehyde 3-phosphate (GAP). Likewise, photosynthetic ATP is needed in the CBB cycle at the steps of phosphoglycerate kinase (Pgk) and phosphoribulokinase (Prk). Most of the newly synthesized organic carbon is used as building blocks to produce fresh biomass during growth. Excess of fixed organic carbon is funnelled into gluconeogenesis for the formation of glycogen (Gründel et al., 2012), which can be also regarded as a metabolic and energetic buffer in cyanobacteria (Cano et al., 2018). Under conditions of non-balanced nutrient and energy conditions, the amount of newly fixed carbon compounds can exceed the growth demand and storage capacity, which can then be released into the medium. Such an overflow mechanism releasing mainly the organic acids pyruvate and 2-oxoglutarate has been observed under high C/N and high light conditions (Gründel et al., 2012; Cano et al., 2018).

During gluconeogenesis, the precursor metabolite GAP is enzymatically converted to glucose 1-phosphate (G1P), which includes a key reaction by fructose 1,6-bisphosphatase (FBPase) to convert fructose 1,6-bisphosphate (FBP) to fructose 6-phosphate (F6P), a metabolite that is shared with the CBB cycle. Glycogen is a glucose polymer and serves as a storage compound in cyanobacteria (Ball and Morell, 2003; Zilliges, 2014). Its synthesis starts with the formation of ADP-glucose from G1P and ATP by a G1P adenylyl-transferase (GlgC). The glycogen synthase GlgA transfers the glycosyl group from ADP-glucose to an existing glycogen chain by an α-1,4 glycosidic bond. Branching is then possible through insertion of α-1,6 glycosidic bonds by the glycogen branching enzyme GlgB (Preiss and Romeo, 1994).

#### 1.1.5 Catabolic carbon routes and glycogen breakdown

Glycogen degradation can provide organic carbon and energy in conditions when Ci fixation is insufficient for energetic and metabolic needs (Preiss and Romeo, 1994). First, glycogen chains are enzymatically debranched and split by debranching enzyme GlgX and glycogen phosphorylase GlgP to release glucose or G1P, which are then converted to glucose 6-phosphate (G6P) by hexokinase and phosphoglucomutase (Pgm), respectively (Doello et al., 2018). The glucose can also originate from uptake. G6P is the key metabolite for catabolic carbon metabolism as it is the shared entry point of three main routes: Embden-Meyerhof-Parnas (EMP) pathway, oxidative pentose phosphate (OPP) pathway, and possibly Entner-Doudoroff (ED) pathway (Chen et al., 2016). Along these pathways, C6 sugars like glucose are stepwise oxidized to the C3 intermediate GAP that can then enter lower glycolysis. The enzymatic reactions of the lower glycolysis converting GAP to pyruvate are shared between all routes. The oxidative reactions of these catabolic pathways are coupled to the production of ATP and different ratios of NADH and/or NADPH (Vermaas, 2001).

The EMP pathway, also called “classical” glycolysis, is the energetically most efficient route producing two molecules of ATP per molecule glucose. The key enzyme phosphofructokinase (Pfk) catalyses the conversion of F6P to FBP. It is encoded by two gene copies in the *Synechocystis* genome (Knowles and Plaxton, 2003).

The OPP pathway is regarded as the main route for glucose catabolism in cyanobacteria. Many reactions run in the opposite direction of the regenerative phase of the CBB cycle thus producing C5 sugars essential for biosynthesis of nucleic acids (Ueda et al., 2018). It is the only way for NADPH production in the dark and requires lower enzymatic resource cost due to shared enzymes with the CBB cycle (Yang et al., 2002). The OPP pathway key enzymes are the glucose 6-phosphate dehydrogenase (Zwf, G6PDH) and the 6-phosphogluconate dehydrogenase (Gnd), which catalyse the oxidation of G6P to 6-phosphogluconate (6PG) and then the oxidative decarboxylation to ribulose 5-phosphate (R5P), respectively. The intermediate F6P is shared with the CBB cycle and the EMP pathway and thus allows the OPP pathway to be either cyclic or non-cyclic (**Figure 1**). In the cyclic mode, F6P from the OPP pathway is converted back to G6P to re-enter the cycle, whereas in the non-cyclic mode F6P is metabolized by Pfk to enter glycolysis.

The ED pathway is the latest discovered glycolytic route in cyanobacteria (Chen et al., 2016). It branches off the OPP pathway with a 6-phosphogluconate dehydratase (Edd) producing the intermediate 2-dehydro-3-deoxy-6-phosphogluconate (KDPG) from 6PG. The key enzyme Eda, an KDPG aldolase, directly converts KDPG to GAP and pyruvate. This route does not share intermediates with the CBB cycle and was thus proposed as the preferential glycolytic route in conditions of photosynthetic CO_2_ assimilation (Chen et al., 2016). However, its contribution to the overall glucose degradation has recently been debated (Bachhar and Jablonsky, 2022; Schulze et al., 2022) and its occurrence in the majority of cyanobacteria is questionable (Evans et al., 2024).

There are also increasing indications that, in addition to the three above mentioned glucose-degrading routes, the phosphoketolase (Pkt) reaction(s) can play an important role in cyanobacteria. Pkt’s are thiamine-pyrophosphate-dependent enzymes that cleave xylulose 5-phosphate or fructose 6-phosphate to acetyl-phosphate and glyceraldehyde 3-phosphate or erythrose 4-phosphate, respectively, without releasing any CO_2_. The *Synechocystis* genome encodes for two Pkt isoenzymes, Pkt1 (*slr0453*) and Pkt2 (*sll0529*) (Bachhar and Jablonsky, 2020), while only one Pkt exists in *Synechococcus elongatus* PCC 7942 (Chuang and Liao, 2021). Despite the presence of Pkt-encoding genes in many cyanobacterial genomes, the role of these enzymes for cyanobacterial metabolism initially remained elusive. Meanwhile it has been shown that Pkt in *Synechocystis* is necessary for the utilization of external xylose (Xiong et al., 2015) and a *pkt* mutant of *S. elongatus* PCC 7942 was unable to survive under dark anaerobic conditions (Chuang and Liao, 2021). Moreover, the Pkt from the latter cyanobacterial strain is regulated by ATP and was shown to play an important regulatory role to balance CO_2_ fixation and energy level under varying growth conditions (Lu et al., 2023).

In addition to the glucose degradation, organic carbon can be directly funnelled into lower glycolysis from the CBB cycle via the conversion of 3PGA to 2PGA by phosphoglycerate mutase (Pgam), especially under low C/N ratios. Pyruvate is mainly converted to acetyl-CoA and channelled into the tricarboxylic acid (TCA) cycle producing 2-oxoglutarate (2OG) as acceptor for ammonia assimilation or fatty acid biosynthesis pathways (Zhang and Bryant, 2011). Another main sink of acetyl-CoA is the biosynthesis of PHB (Hauf et al., 2013), which is the second cyanobacterial carbon and energy storage compound and accumulates highest under long-term nitrogen or phosphate starvation (Koch et al., 2020).

#### 1.1.6 Glycolytic routes as anaplerotic shunts

In photoautotrophic conditions, the central carbon metabolism in cyanobacteria is mainly associated with the fixation of CO_2_ in the CBB cycle for generation of carbon skeletons, and rerouting excess fixed carbon towards glycogen formation. During the last years, the image of the CBB cycle as an autonomous, self-sufficient cycle has been rated an oversimplified view. Instead, the CBB cycle is embedded into a network of replenishing and consuming reactions to ensure continuous operation, similar to the TCA cycle. Makowka et al. (2020) analysed these replenishing properties of the ED, EMP, and OPP pathways in *Synechocystis*. Their function as anaplerotic shunts for the CBB cycle in light was initially indicated by a decreased photoautotrophic growth and impaired glycogen catabolism in light for *Synechocystis* mutant strains deficient in the KDPG aldolase Eda. Follow-up research confirmed the importance of glycolytic shunts to restart an arrested CBB cycle after dark to light transitions (Makowka et al., 2020). These catabolic routes thereby fine-tune CO_2_ fixation and carbon allocation in *Synechocystis* under photoautotrophic conditions. Recent flux analyses showed that the anaplerotic reactions branching off the EMP pathway forming the so-called phosphoglucose isomerase (PGI) shunt contributed most to refill the CBB cycle followed by the OPP shunt, whereas no carbon flux could be measured through the ED pathway and Pkt under mixotrophic conditions (Schulze et al., 2022).

#### 1.1.7 The triose-phosphate hub around key enzymes GapDH and Pgam

**Figure 2:**
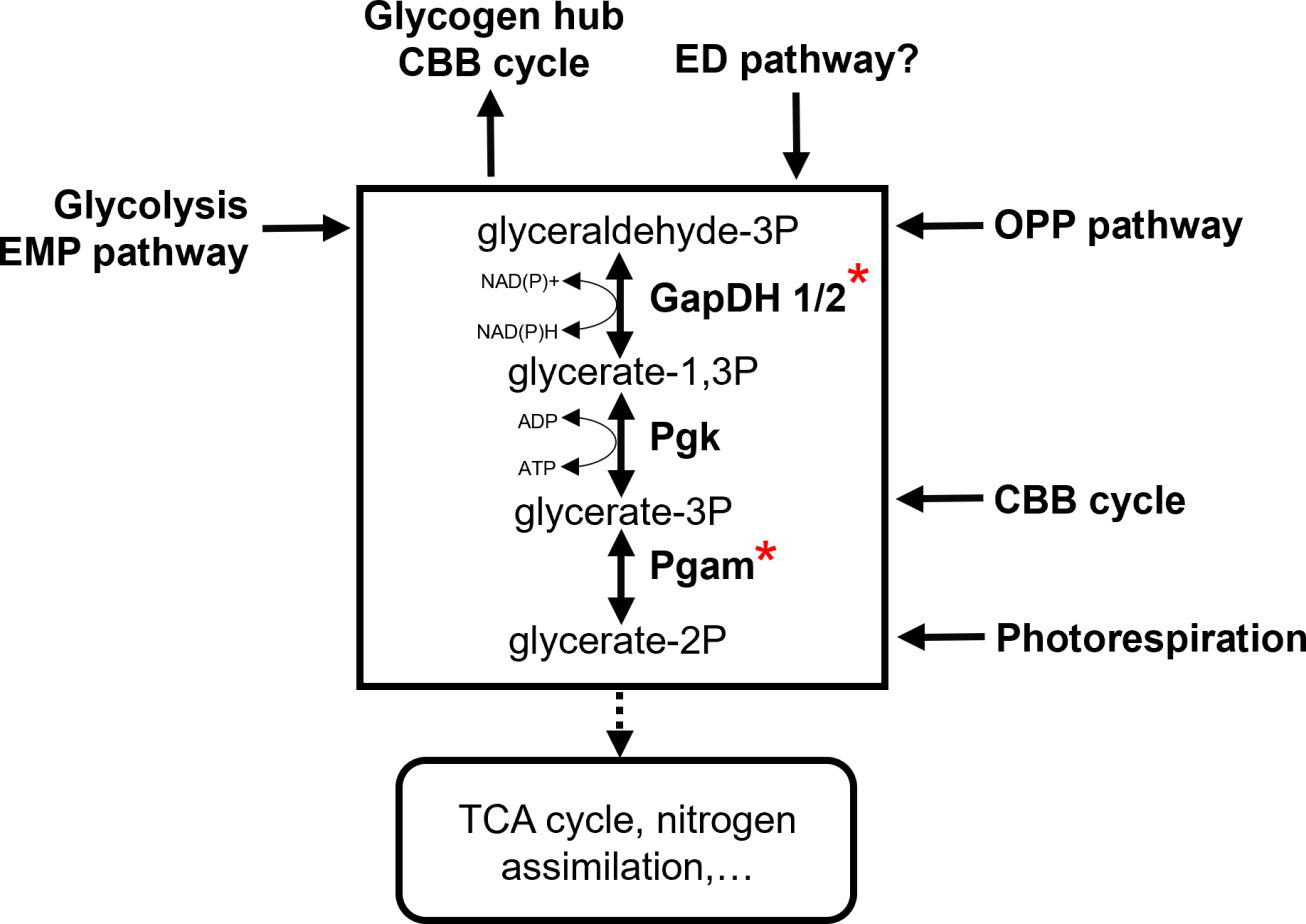
The central role of triose-phosphate metabolism for the primary carbon metabolism in the non-compartmented cyanobacterial cell. (GapDH, Glyceraldehyde 3-phosphate dehydrogenase; Pgam, Phosphoglycerate mutase; Pgk, Phosphoglycerate kinase; red asterisks mark phosphoproteins).

All metabolic routes involved in primary carbon metabolism merge at the interconversion of triose-phosphates (**Figure 2**). Hence, the enzymatic reactions and intermediates ranging from the stepwise conversion of GAP to 2PGA and *vice versa* in the lower glycolysis have been identified as a central carbon distribution hub, i.e. the “triose-phosphate hub”. This hub contains key enzymes GapDH and Pgam as major branching points for organic carbon allocation. Therefore, it serves as an important target for metabolic regulation of the autotrophy-heterotrophy switch in *Synechocystis*, which requires coherent decision-making and precise regulation of metabolic fluxes for two reasons. First, fluxes need to be directed depending on the environmental conditions like light intensity and extracellular Ci concentrations. In high CO_2_ conditions, gluconeogenesis can be enhanced to accumulate glycogen as storage compound (Eisenhut et al., 2008b; Gründel et al., 2012). In low light and Ci limitation, the CBB cycle provides fewer GAP, and anaplerotic reactions in catabolic direction are preferred. Second, cyanobacterial GapDH and Pgam can catalyse the reactions in the anabolic as well as catabolic directions (Koksharova et al., 1998; Jablonsky et al., 2013). Without regulatory mechanisms, these enzymes would cause futile reactions halting the directed carbon allocation, and wasting energy in the process.

There are two GapDH isoenzymes in *Synechocystis* with distinct functions (Koksharova et al., 1998). GapDH1 is a protein of 354 amino acids (37.8 kDa) encoded by the gene *slr0884*. GapDH2, encoded by *sll1342*, contains 337 amino acids (36.4 kDa). Both are bidirectional enzymes that catalyse the interconversion of GAP and G1,3BP in the triose-phosphate hub. However, they diverge in the use of coenzymes. While GapDH2 can utilise both NAD(H) and NADP(H), GapDH1 is only able to use NAD(H). Therefore, GapDH2 is mainly part of the CBB cycle, in which it reduces G1,3BP using photosynthetic NADPH (Koksharova et al., 1998). The catabolic reactions of both GapDH variants are crucial for heterotrophic and mixotrophic growth using stored glycogen or external glucose, respectively. Two Pgam isoenzymes are annotated for *Synechocystis*. Protein Slr1945 (Pgam1) is a 2,3-bisphosphoglycerate-independent phosphoglycerate mutase, while putative Pgam2 Slr1124 has an additional annotation as phosphoserine phosphatase (Klemke et al., 2015). In addition to the existence of GapDH and Pgam isoenzymes, both GapDH1 and 2 as well as Pgam1 have been identified as phosphoproteins in *Synechocystis* under specific conditions (Spät et al., 2023), which could provide an additional layer of carbon flux regulation.

#### 1.1.8 Carbon metabolism in changing CO_2_ conditions

Glycogen turnover is not bound to day-night changes. Fluctuating glycogen levels in response to changing Ci and N availability have been reported for *Synechocystis* in constant light (Doello et al., 2018; Lucius et al., 2021) and photosynthetic fluxes via the OPP pathway into the CBB cycle have been measured (Young et al., 2011). *Synechocystis* cells grown in high CO_2_ conditions accumulate high levels of glycogen, whereas they mobilize stored glycogen when carbon supply is limited (Eisenhut et al., 2008b). A sudden decrease of external Ci levels requires a rapid acclimation of the cyanobacterial metabolism for carbon assimilation and catabolic glycolytic routes. In that, carbon is drained from the CBB cycle via glycolysis and TCA cycle to maintain biosynthesis of amino acids while photorespiration is activated (Jablonsky et al., 2016). This condition can be simulated by CO_2_ shift experiments. To this end, *Synechocystis* cultures grown with supplied 5% CO_2_ (high CO_2_ conditions, HC) are shifted to ambient air conditions with 0.04% CO_2_ (low CO_2_ conditions, LC) by manually abruptly cutting off the excess CO_2_ aeration. Such CO_2_ shift protocols have been established to study CO_2_-dependent changes of the central metabolism, combined with metabolite and transcript analyses in continuous light. Previous studies revealed that *Synechocystis* cells undergo a global metabolic reprogramming upon HC to LC shifts (Eisenhut et al., 2008b) with a distinct metabolic signature that is similar to metabolic changes in *Arabidopsis thaliana* under low versus high photorespiratory flux (Orf et al., 2016b). Here, CBB cycle activity decreases while the accumulation of metabolites like 2PG, glycine and a transient increase of 3PGA point to an active photorespiration. Simultaneously, glycogen breakdown is enhanced, and most amino acid levels decline (Orf et al., 2016b). Interestingly, shifts from HC to LC do not cause significant changes in transcript levels for enzymes involved in primary carbon metabolism (Klähn et al., 2015). Likewise, proteomic studies revealed that carbon metabolism proteins respond more strongly to light changes, but barely to different Ci availability (Jahn et al., 2018; Spät et al., 2021; Barske et al., 2023). Such findings point toward biochemical control rather than transcriptional regulation to enable a quick acclimation of carbon partitioning without comparatively high energetic costs for proteomic responses (Jablonsky et al., 2016).

### 1.2 Connection of carbon and nitrogen metabolism

#### 1.2.1 Nitrogen assimilation in cyanobacteria

Apart from carbon, nitrogen is needed for cyanobacterial growth and is incorporated into essential cellular building blocks (Esteves-Ferreira et al., 2018). Most cyanobacteria including *Synechocystis* are non-diazotrophic strains and rely on combined N sources such as nitrate (NO_3_^-^) or nitrite (NO_2_^-^), which are taken up by the ABC transporter NrtABCD (Luque et al., 1994; Aichi et al., 2001). Intracellular nitrate is then converted to NH_4_^+^ via nitrate reductase and nitrite reductase, the latter enzyme is regulated in response to Ci shifts by a recently identified small regulatory protein NirP1 (Kraus et al., 2024). Direct uptake of NH_4_^+^ is achieved via ammonium permeases under low nitrogen conditions, while in the presence of high ammonia, this nutrient can diffuse through the cytoplasmic membrane (Montesinos et al., 1998). Cellular NH_4_^+^ is incorporated into the primary metabolism via the glutamine synthetase/glutamine oxoglutarate aminotransferase (GS/GOGAT) cycle (Muro-Pastor et al., 2005). In this cycle, GS first catalyses the reaction of glutamate and NH_4_^+^ to glutamine at the expense of ATP. The amino group of glutamine is then transferred to 2OG by GOGAT using reduced ferredoxin, generating two molecules of glutamate (Herrero et al., 2016). One glutamate molecule can re-enter the cycle while the second one can be directed towards further biosynthetic pathways.

In contrast to other bacteria, cyanobacteria adjust their metabolism according to their internal homeostasis, rather than directly reacting to environmental changes (Galperin, 2005). Therefore, it is essential to monitor intracellular metabolic levels to readjust cyanobacterial carbon to nitrogen (C/N) balance to a C/N ratio of approximately 5:1 (Wolk, 1973). As mentioned above, the carbon metabolism is directly linked to the N metabolism via 2OG. The production of this carbon precursor for N assimilation is one of the main functions of the “open” TCA cycle in cyanobacteria (Steinhauser et al., 2012). The TCA cycle is fuelled by CO_2_ fixation products via lower glycolysis and from there, 2OG enters the GS/GOGAT cycle. The availability of 2OG as a carbon skeleton essentially influences the rate of N assimilation (Esteves-Ferreira et al., 2018). Changing 2OG levels signalize changes in the C/N balance. While assimilation of ammonium reduces the 2OG pool, carbon allocation towards the TCA cycle leads to increased levels. Likewise, an accumulation of 2OG signalizes a limitation of N sources and a C/N imbalance towards carbon. These metabolic signals are mainly monitored by the regulatory protein PII in cyanobacteria as well as in chloroplasts of plants.

#### 1.2.2 Regulation of N metabolism by the PII protein

The trimeric PII protein family is highly conserved among cyanobacteria, heterotrophic bacteria, and plants (Forchhammer et al., 2022). These proteins can integrate metabolic signals by binding specific effector molecules. In general, this binding causes conformational changes in the protein structure, which enables PII proteins to interact with various targets to maintain the C/N homeostasis. In *Synechocystis*, ATP and ADP can bind to PII communicating the energy state of the cell. When Mg^2+^-ATP is already bound, PII can additionally bind 2OG reporting the relative C/N ratio. The bound metabolites cause conformation changes in the target-binding loop structures (T-loops) specific for the effector molecule, and thereby enable specific interaction with a range of target proteins (Merrick, 2015).

In cyanobacteria, PII can target different enzymes such as the N-acetyl-L-glutamate kinase (NAGK) that controls arginine biosynthesis (Heinrich et al., 2004). During low 2OG levels due to sufficient N sources, PII with bound ATP can form an activating complex with NAGK leading to a higher flux towards arginine (Lüddecke and Forchhammer, 2015). Arginine can then be utilised for the formation of cyanophycin, the cyanobacterial N storage compound (Stephan et al., 2000). High 2OG levels lead to the dissociation of the PII-NAGK complex (Maheswaran et al., 2004). In excess N conditions, PII can also bind to the small protein PipX, a coactivator of NtcA, the global cyanobacterial N control transcription factor (Espinosa et al., 2007). At higher 2OG levels indicating N depletion, the PII-PipX complex dissolves and PipX can bind to NtcA again to regulate expression of N assimilation related genes (Espinosa et al., 2014). An example how PII can additionally regulate the carbon metabolism is given by its interaction with phosphoenolpyruvate carboxylase (PEPC). PEPC is the second major enzyme for Ci fixation in *Synechocystis* and is activated by PII-ATP in the presence of 2OG (Scholl et al., 2020).

## 2 Regulation of the carbon metabolism during changing carbon supply

For planktonic cyanobacteria such as *Synechocystis*, the ever-changing location in the waterbody influences light availability and solubility of CO_2_, thus causing fluctuations in Ci and energy supply (Badger et al., 2006). These environmental changes require a flexible and equally rapid switch between different growth modes to adjust its metabolism according to resource availability, as anabolic and catabolic carbon metabolism share intermediates and enzymes (Makowka et al., 2020). The different trophic modes are accompanied by a different carbon allocation/partitioning, i.e., directing organic carbon for example into gluconeogenesis or into lower glycolysis for N assimilation. The defined routing of intermediates requires a complex decision process based on internal cellular factors like growth stage, metabolite values, glycogen disposability, and external factors like relative concentrations of Ci and N sources (Ciebiada et al., 2020).

There are multiple strategies for biochemical regulation of enzymes in the central carbon metabolism to adjust carbon allocation in changing Ci levels. These strategies include protein-protein interaction, protein phosphorylation, redox regulation, signalling cascades, or effector molecule binding. These mechanisms can inhibit or enhance enzymatic activity at branching points temporarily without affecting the respective protein abundance (Jablonsky et al., 2016). For example, the isoenzymes in the triose-phosphate hub, Pgam1, Pgam2, GapDH1 and GapDH2 need to be tightly regulated to enable the adjustment of carbon allocation in anabolic or catabolic direction for *Synechocystis* during acclimation to varying Ci or light conditions (Koksharova et al., 1998; Jablonsky et al., 2014). This can be achieved via protein modification such as reversible protein phosphorylation of Pgam1, GapDH1 and/or GapDH2. Previous studies revealed alterations in the *Synechocystis* phospho-proteome depending on CO_2_ availability for important carbon-related proteins like Cp12 and BCT1 subunits (Spät et al., 2021; Barske et al., 2023). In the glycogen turnover, crucial phosphorylation sites of the key enzyme Pgm1 for glycogen mobilization were discovered (Doello et al., 2022). Redox regulation is another way of adjusting the metabolism to changing Ci conditions. Reducing equivalents like NADPH are produced by the photosynthetic electron transport chain and are utilised for reductive reactions of the CBB cycle. Therefore, a strategy to adjust carbon allocation is to sense the cellular redox status (Ansong et al., 2014). Key enzymes in the CBB cycle of plants like Prk and GapDH are redox-regulated in a thioredoxin-mediated way, whereas the majority of cyanobacterial CBB cycle enzymes are not directly redox-regulated (Lindahl and Florencio, 2003; Tamoi et al., 2005). During the last 5 years, new aspects of the regulatory network appeared that will be explained in the following.

### 2.1 The potential regulatory role of the Eda enzyme on carbon metabolism

**Figure 3:**
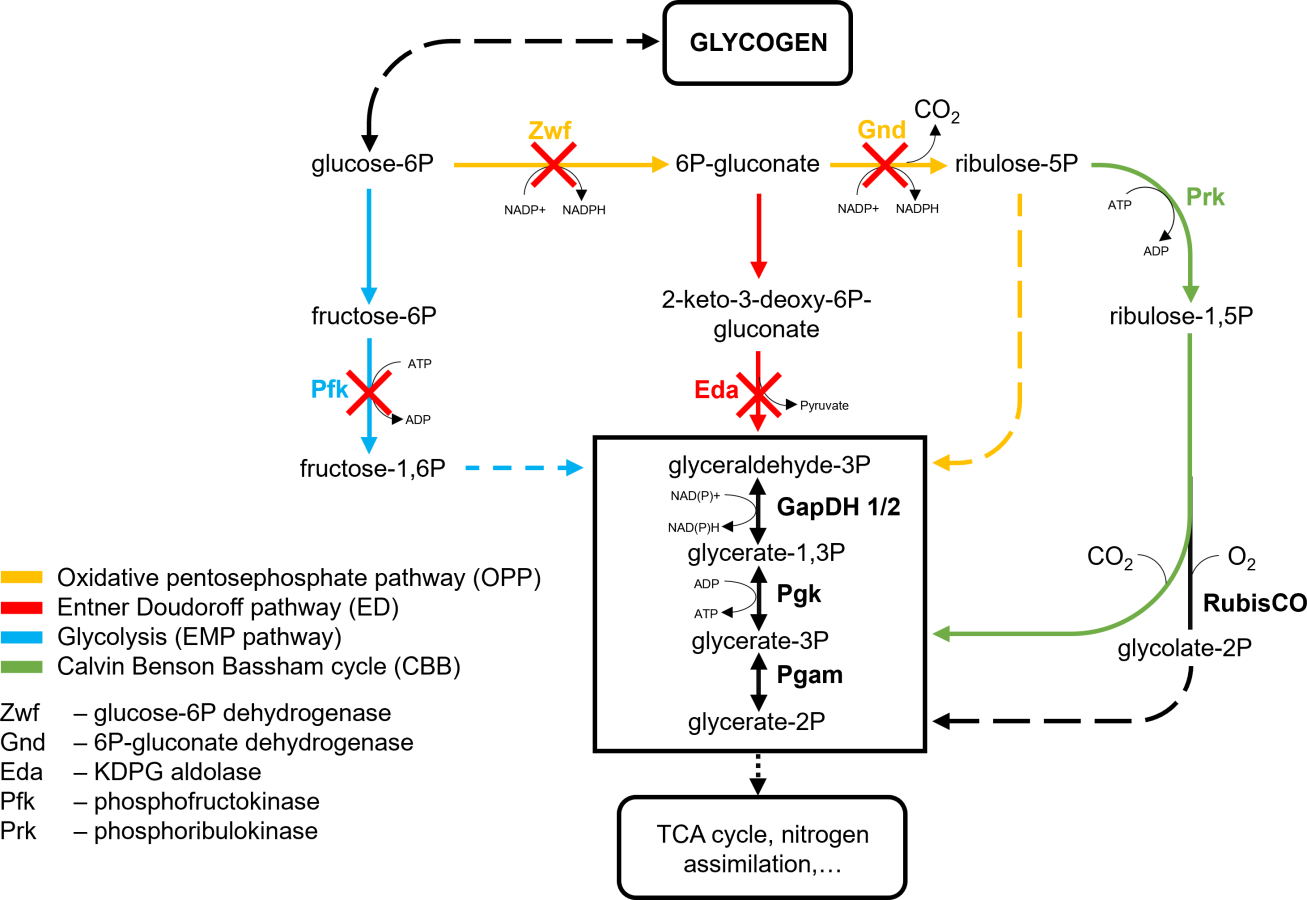
Central carbon metabolism of *Synechocystis* with indication of affected enzymes in the studied mutant strains. Adapted from Lucius et al. (2021). GapDH, Glyceraldehyde 3-phosphate dehydrogenase; Pgam, Phosphoglycerate mutase; Pgk, Phosphoglycerate kinase; RubisCO, Ribulose 1,5-bisphosphate carboxylase/oxygenase.

The existence of different routes for glucose catabolism could indicate that they may play specific roles in carbon partitioning under specific environmental conditions. Especially the discovery of the ED pathway in cyanobacteria offered the attractive possibility that this catabolic route could work in parallel to the anabolic CBB cycle because there is no overlap in enzymes and intermediates. The set of *Synechocystis* deletion mutants generated by Chen et al. (2016) made it possible to investigate the specific roles of the glycolytic ED and EMP routes as well as the OPP pathway. By knocking out the gene for the *Synechocystis* G6PDH/Zwf, the mutant Δ*zwf* has OPP as well as ED pathways blocked. Only the OPP pathway is blocked in Δ*gnd* after deletion of the gene for the 6-phosphogluconate dehydrogenase Gnd. The mutant Δ*pfk* has the genes for both isoforms of the phosphofructokinases PfkB1 and PfkB2 deleted, thus interrupting the EMP pathway. In the mutant Δ*eda*, the gene for the KDPG aldolase Eda encoded by *sll0107* is knocked out to specifically disrupt the ED pathway in *Synechocystis* (**Figure 3**).

This mutant set was analysed regarding differences in growth and glycogen turnover under different trophic conditions (Chen et al., 2016), after awakening of dormant cyanobacteria from N starvation induced chlorosis (Doello et al., 2018), under light/dark transition (Makowka et al., 2020), and upon changing Ci conditions (Lucius et al., 2021). All these studies provided evidence that the ED pathway might be of particular importance, because *Synechocystis* mutant strains deficient in the KDPG aldolase Eda showed decreased photoautotrophic and delayed mixotrophic growth with impaired glycogen catabolism in the light (Chen et al., 2016). Follow-up research confirmed the importance of glycolytic shunts to restart an arrested CBB cycle after dark to light transitions (Makowka et al., 2020). Deficiencies in glycogen breakdown in mutant Δ*eda* also delayed the awakening of dormant cells upon N supply (Doello et al., 2018).

The behaviour of these strains during shifts from HC to LC and back to HC conditions in continuous light was analysed by Lucius et al. (2021). As expected, the wild type showed high glycogen levels under HC conditions, when excess organic carbon can be stored. Glycogen rapidly decreased in the wild type within 24 hours after the LC shift, representing its quick mobilization to rebalance carbon fluxes in the central carbon metabolism towards the TCA cycle and downstream cellular biosynthesis pathways. This was accompanied by a simultaneous growth delay. After the subsequent shift back to HC levels, wild-type cells rapidly accumulated glycogen again as increased CO_2_ fixation promotes gluconeogenesis. Cells resumed growth within 24 hours after the second shift. Basically similar alterations were observed for Δ*zwf*, Δ*gnd* and Δ*pfk* during the HC-LC-HC shifts, whereas the mutant Δ*eda* showed a significantly different pattern in cellular glycogen amounts (Lucius et al., 2021). Initial levels were around two times higher compared to all other examined *Synechocystis* strains. Therefore, the synthesis of glycogen in HC conditions is not impaired when Eda is missing. After the HC-LC shift, glycogen was consumed comparatively slowly. However, most notably, glycogen levels did not increase again in response to the LC-HC shift but stayed stable for at least three hours afterwards. Furthermore, after 24 hours under resupplied HC conditions, Δ*eda* showed a significant growth delay compared to the other strains. These results seemed to indicate that apparently, *Synechocystis* relies on the glycolytic ED shunt or Eda activity to replenish and thus reactivate the CBB cycle after Ci limitation periods due to glycogen consumption, as discovered by Makowka et al. (2020). Altogether, an intact Eda enzyme supports the rapid reactivation of growth by elevated Ci levels after periods of LC, which cannot be compensated by EMP and OPP pathways.

On the first view, these findings seemed to indicate that the ED pathway makes a significant contribution to sugar catabolism in the light. However, this interpretation is hampered by contradicting observations. In experiments analysing the mutant set under fluctuating Ci conditions, the mutant Δ*zwf* showed basically wild-type-like responses of growth and glycogen pools at the different Ci levels (Lucius et al., 2021). In this mutant, the G6PDH is completely absent, which should block carbon flow not only into the OPP but also into the ED pathway. Hence, the different responses of the mutants Δ*zwf* and Δ*eda* make it unlikely that a block of the carbon flux through the ED pathway is the correct explanation for the observed differences in glycogen pools of these two mutants. Recently, Schulze et al. (2022) reported that no flux through the ED pathway towards KDPG could be detected using GC-MS-based ^13^C-based metabolic flux analyses under different trophic conditions, while substantial carbon flow through the EMP glycolysis and the OPP pathway was measured. This experimental paper (Schulze et al., 2022) is supporting bioinformatic predictions of rather low contributions of the ED pathway to overall carbon flux at least under steady state conditions (Bachhar and Jablonsky, 2022). These results indicate that Eda might operate on another, most likely regulatory layer apart from different metabolic activity.

In addition to the analysis of glycogen levels and growth under changing Ci conditions, the metabolism was analysed in these *Synechocystis* strains, especially amino and organic acids as well as the RuBisCO products 3PGA and 2PG. In the wild type, the two latter metabolites accumulate during LC acclimation because of increased oxygenation reaction of RuBisCO (2PG) and a decreased metabolization during readjustment of the CBB cycle (3PGA). Mutant strains Δ*zwf*, Δ*gnd* and Δ*pfk* accumulated 3PGA and 2PG amounts comparable to the *Synechocystis* wild type. An ED pathway, which is blocked at the Eda step, suggests a preference for the OPP shunt as a replacement. Here, release of CO_2_ at the Gnd decarboxylation step might reduce photorespiration in low Ci conditions. Such a blockage would also prevent replenishment of the CBB cycle with triose-phosphates as Eda also plays a role in glycogen breakdown in low CO_2_ conditions. These implications are supported by distinctly lower accumulations of 2PG and 3PGA in Δ*eda* after the HC-LC shift in comparison with the wild type. In addition, several amino acids changed in Δ*eda.* Particularly, it accumulates high amounts of proline and arginine during LC acclimation (Lucius et al., 2021). As proline accumulation has been reported for maintenance of a redox balance in case of elevated NADPH levels in other organisms (Fichman et al., 2015; Zhang and Becker, 2015), we initially assumed that its increase in Δ*eda* is correlated with the stressed phenotype of this mutant (Lucius et al., 2021). Another possibility is a metabolite spill over from accumulated arginine in response to the first LC shift, as a conversion from arginine to proline via ornithine by AgrE has been reported for cyanobacteria (Zhang and Yang, 2019). Moreover, Eda also has an annotated function as (4S)-4-hydroxy-2-oxoglutarate aldolase (Uniprot database KEGG, entry Q55872_SYNY3; https://www.uniprot.org/uniprotkb/Q55872/entry), which can combine glyoxylate and pyruvate to 4-hydroxy 2-oxoglutarate or catalyse the reverse reaction. Thus the enzyme also participates in amino acid metabolism. This enzymatic activity correlates with the observed strong over-accumulation of proline in the mutant Δ*eda.* Hence, the observed changes in growth and glycogen metabolism could also indirectly result from an imbalanced C/N ratio due to the absence of the (4S)-4-hydroxy-2-oxoglutarate aldolase activity of Eda.

The miracle of the possible occurrence and role of the ED pathway was recently reinvestigated by Evans et al. (2024). This study clearly shows that the ED pathway is absent from the majority of cyanobacteria including *Synechocystis*, because the annotated Edd enzyme cannot convert 6-phosphogluconate into KDGP, but is instead a specific dihydroxy-acid dehydratase (DHAD) acting exclusively in the branched chain amino acid pathway. However, Eda homologs from cyanobacteria were proven as biochemical active aldolases with multiple substrate specificities (Evans et al., 2024). Hence, it is likely that the mutation of *eda* in *Synechocystis* abolishes a moonlighting aldolase reaction leading to metabolic imbalances that are somehow sensed and result in changed glycogen metabolism and other alterations observed in the above mentioned studies.

### 2.2 The Cp12 protein as regulator of the CBB cycle

One important regulatory protein is the conditionally disordered protein Cp12, which is conserved in almost all oxygenic phototrophs, from cyanobacteria to algae and plants. The disordered state is considered the inactive, reduced form of Cp12 while under certain oxidizing conditions, it takes structure mediated by conserved cysteine pairs (Graciet et al., 2003). Canonical Cp12 proteins possess two cysteine pairs close to the N-terminus and C-terminus, respectively, that can form redox-dependent disulphide bridges (Groben et al., 2010). These conformational changes then allow the initially disordered Cp12 protein to structure and subsequently to interact with its main binding partners in the CBB cycle, phosphoribulokinase (Prk) and glyceraldehyde 3-phosphate dehydrogenase (GapDH). Upon reducing conditions, the complex dissociates by disulphide reduction allowing GapDH and Prk to be reactivated along with the CBB cycle. Hence, under dark, oxidized conditions, Cp12 binds Prk and GapDH in a supramolecular ternary complex, thereby inhibiting both enzymes. While Prk and GapDH are also directly redox-regulated by thioredoxins in plants, most cyanobacterial CBB cycle enzymes are not (Tamoi et al., 2005; Marri et al., 2009). Instead, the cyanobacterial redox-regulation of the CBB cycle is mainly done via Cp12, where Cp12 complex formation is mediated in response to the intracellular NAD(H)/NADP(H) ratio (Tamoi et al., 2005).

**Figure 4:**
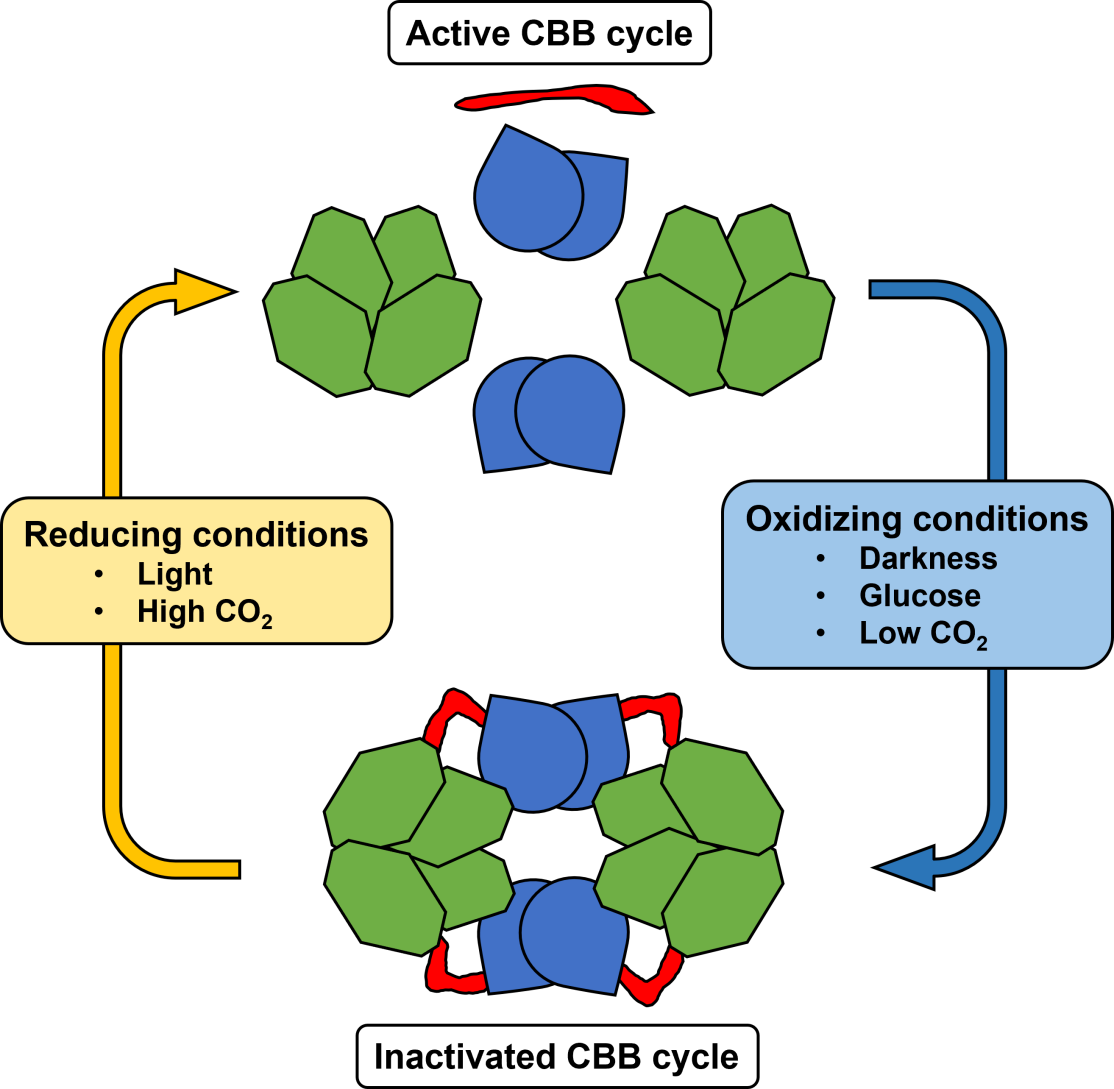
Model of the redox-dependent complex formation and CBB cycle regulation via Cp12-mediated (red) binding of the tetrameric glyceraldehyde 3-phosphate dehydrogenase (GapDH, light green) and the dimeric phosphoribulokinase (PRK, light blue) in cyanobacteria.

The work of McFarlane et al. (2019) solved the crystal structure of the ternary complex between Cp12, Prk, and GapDH in *Thermosynechococcus elongatus*. The cyanobacterial GapDH tetramer is first bound by two oxidized molecules of Cp12 to form a GapDH-Cp12 subcomplex. Subsequently, Cp12 forms an ordered disulphide-locked helical hairpin that allows interaction with Prk (McFarlane et al., 2019). Two GapDH-Cp12 subcomplexes in turn can then bind two Prk dimers, thus forming a ternary supramolecular complex consisting of two GapDH tetramers, two Prk dimers and four ordered Cp12 (**Figure 4**). The canonical consensus sequence AWD_VEEL of Cp12 interacts with the Ru5P-binding site of Prk (Yu et al., 2020). This way, Cp12 molecules bridge the active sites of GapDH and Prk, thereby locking this inhibitory complex (McFarlane et al., 2019).

#### 2.2.1 Cp12-dependent regulation in changing light conditions

Initially, the role of cyanobacterial Cp12 was investigated in the model strain *Synechococcus elongatus* PCC 7942 (hereafter *Synechococcus* 7942). These studies revealed that in addition to serving as a dark-light redox switch of the CBB cycle (Tamoi et al., 2005), Cp12 is involved in high light protection in *Synechococcus* 7942 (Tamoi and Shigeoka, 2021). However, its Cp12 sequence lacks the N-terminal pair of cysteine residues (Tamoi et al., 2005). In *Synechocystis*, the gene *ssl3364* encodes a Cp12 protein of 74 amino acids (8.3 kDa), which possesses both canonical cysteine pairs at the N-terminus (Cys19, Cys29) and C-terminus (Cys60, Cys69), and contains the canonical core sequence AWD_VEEL. Furthermore, the *Synechocystis* CP12 has been identified as phosphoprotein under different CO_2_ conditions, which might offer additional regulatory possibilities (Spät et al., 2021).

*In vivo* experiments can elucidate details about true functions of regulatory mechanisms and provide another dimension of cellular response analyses to actual environmental changes. *Synechocystis* strains expressing eYFP-tagged versions of GapDH2 or Prk, respectively, were used to visualize for the first time the chronology of Cp12-GapDH-Prk complex formation by fluorescence microscopy imaging (Lucius et al., 2022). Clear aggregates visible as fluorescent spots formed in cells of the eYFP strains upon darkness. Signals became first visible in the eYFP-GapDH cells and appeared time-displaced in eYFP-Prk cells. The temporal sequence of this spot formation thus confirms the primary binding of GapDH2 by Cp12 in dark/oxidizing conditions and the subsequent incorporation of Cp12-bound Prk into the Cp12-GapDH-Prk complex. Upon re-illumination, the fluorescent spots dispersed first in the eYFP-Prk strain and then in cells with eYFP-tagged GapDH2. This dissociation resembles the reverse sequence of complex formation. The fluorescent signals appeared as roughly three to five bright circular spots per cell in the eYFP-tagged strains. The reasons for this pattern and potential influence of other cellular mechanisms on the complex localization remain unknown thus far. According to the number of spots formed in darkness, an association of complex formation close to carboxysomes might be possible.

Measurements of the *in vivo* oxidation level of NAD(P)H revealed an importance of Cp12 during redox state regulation dependent on light intensity (Lucius et al., 2022). This was assessed by comparing the NAD(P)H fluorescence in wild type and Δ*cp12* cells exposed to different light intensities and during a subsequent high light pulse followed by an immediate shift to darkness. The high light pulse reduces the major part of the photo-reducible NAD(P)^+^ pool to NAD(P)H. The rate of re-oxidation of NADPH to NADP^+^ after the dark shift thus depends on the level of CBB cycle regulation during the light acclimation before the light pulse, because uninhibited GapDH2 would quickly oxidize NADPH in darkness via its respective anabolic reaction. In fact, the experiment suggests a gradually increased association of Cp12 with GapDH2 in decreasing light intensities. Therefore, the CBB cycle is not only downregulated by Cp12 in dark/light phases but also fine-tuned in lower light intensities and fluctuations thereof. This is indicated by a faster NAD(P)H oxidation rate in darkness for the wild type the higher the pre-acclimation light intensity was set. During acclimation to lower light, the CBB cycle is gradually downregulated by partial Cp12-mediated binding of GapDH2 and thus the re-oxidation rate is lower in wild type cells. The crucial role of Cp12 for this re-oxidation is confirmed by redox measurements on the Cp12-deficient mutant, which displays consistently high oxidation rates after the dark shift, independent of previous light settings (Lucius et al., 2022).

#### 2.2.2 Cp12-dependent regulation in changing CO_2_ conditions

The CBB cycle activity also depends on availability of Ci. Therefore, potential Cp12 regulation was also analysed during acclimation to changing CO_2_ levels in constant light using the *Synechocystis* wild type and mutant Δ*cp12* (Lucius et al., 2022). The role of Cp12 in Ci acclimation is indicated by results from CO_2_ shift experiments comparing metabolites that are part of the CBB cycle and the central carbon metabolism routes in general. Among them, the levels of the RuBisCO substrate RuBP strongly increased in Δ*cp12* in response to the HC-LC shift compared to the wild type. Similarly, levels of dihydroxyacetone phosphate (DHAP), the more detectable isomer of GAP, increased in the Cp12-deficient strain compared to wild type upon the shift. This points at a more active CBB cycle with increased activities of Prk and GapDH2 due to the lack of Cp12 inhibition, whereas wild type cells reduce CBB cycle activity after a HC-LC shift. The lack of Cp12 does not affect the activity and abundance of GapDH2 and Prk in *Synechocystis* (Blanc-Garin et al., 2022), in contrast to Arabidopsis in which a Cp12-deficiency causes decreased Prk expression (López-Calcagno et al., 2017).

#### 2.2.3 Cp12 regulation during glucose supply

In addition to light/dark or Ci changes, the utilization of external glucose impacts the cellular redox state and perhaps the Cp12-mediated regulation. The connection between redox regulation of Cp12 complex formation and external glucose supply was investigated using a set of *Synechocystis* strains expressing mutated Cp12 versions that are deficient in binding to GapDH2 and/or Prk (Lucius et al., 2022). The wild-type variant of Cp12 harbours two cysteine pairs and thus belongs to the group of Cp12-N/C according to Stanley et al. (2013). The GapDH-binding domain resides at the C-terminus and the Prk-binding domain in proximity to the N-terminus. In addition to the deletion mutant Δ*cp12*, complementation strains were generated expressing variants of Cp12, lacking either the N-terminal cysteines Cys19 and Cys29 (Δ*cp12::cp12*-ΔCysN), or the C-terminal pair by Cys60 and Cys69 (Δ*cp12::cp12*-ΔCysC), along with a strain expressing a Cp12 variant lacking all four cysteines (Δ*cp12::cp12*-ΔCysNC). These strains permit the analysis of the relative importance of Cp12-mediated inhibition of either GapDH2 or Prk in response to glucose addition. However, cyanobacterial Prk also possesses cysteine residues that allow direct redox regulation of this enzyme independent from the Cp12-mediated mechanism (Fukui et al., 2022).

In the light, external glucose can be utilised for glycogen formation or channelled into glycolytic routes thereby replenishing the CBB cycle (e.g., Chen et al., 2016). Hence, glucose in the light should further increase the reduced state and accordingly no growth differences were found between wild type and Δ*cp12* at standard growth light. However, in low light intensities of 12.5 µmol photons m^-2^ s^-1^, mutant Δ*cp12* shows a clearly diminished growth in liquid medium supplied with glucose (Blanc-Garin et al., 2022). This necessity for Cp12 in decreasing light under glucose influence is further confirmed by the inability of Δ*cp12* to grow heterotrophically in darkness on glucose (Lucius et al., 2022). Thus, Cp12 appears crucial for glucose catabolism in low photosynthetic activity, when most of the glucose is catabolized by the OPP pathway. Further, the lack of Cp12 leads to growth inhibition under photoheterotrophic conditions in constant light with added DCMU (3-(3,4-dichlorophenyl)-1,1-dimethylurea) inhibiting PSII activity (Blanc-Garin et al., 2022; Lucius et al., 2022). In the presence of DCMU, the cell mainly relies on reducing power generated from glucose oxidation that is sufficient for the *Synechocystis* wild type but does not work for Δ*cp12* cells. These results show that Cp12-dependent regulation is relevant during other redox imbalances caused by environmental fluctuations in addition to day-night transitions.

In diurnal growth of alternating 12-hour phases of light and darkness without external glucose supply, Δ*cp12* displays a wild-type-like growth and thus, Cp12 regulation appears dispensable under this condition in *Synechocystis* (Blanc-Garin et al., 2022; Lucius et al., 2022). For other cyanobacterial strains such as *Synechococcus* 7942, a Cp12-deficient strain even shows impaired growth after long-term acclimation to diurnal growth (Tamoi et al., 2005). However, when diurnal growth experiments are performed with the *Synechocystis* wild type and the Cp12-deficient strain in the presence of glucose, the Δ*cp12* mutant is unable to grow in alternating 12-hour dark/light phases (Blanc-Garin et al., 2022; Lucius et al., 2022). Further growth experiments in 12-hour diurnal conditions in glucose-containing medium revealed a wild-type-like growth for the strain Δ*cp12::cp12*-ΔCysN impaired in Prk-binding. In contrast, strains Δ*cp12::cp12*-ΔCysC and Δ*cp12::cp12*-ΔCysNC with affected GapDH2 binding show a strong growth impairment comparable to Δ*cp12* with a rather late acclimation to such light and glucose conditions. Hence, inactivation of GapDH2 by Cp12 is essential for dark glucose utilisation from the medium and proper breakdown of glycogen reserves (Lucius et al., 2022).

Glucose metabolism might cause a redox imbalance for the cell in situations where GapDH2 should be inactive. Catabolic production of NADPH from glucose oxidation then could be opposed by anabolic NADPH consumption by GapDH2 in *Synechocystis* leading to futile cycles. In darkness, Zwf and Gnd of the OPP pathway are the only enzymes that produce NADPH from oxidation of glucose. Therefore, the NADPH pool is limited and should only be available for metabolic enzymes that require this reducing equivalent and cannot accept NADH. In low photosynthetic activity in darkness or low light, GapDH1 is the active GapDH isoenzyme which only utilises NADH, while GapDH2 is inhibited by Cp12. This inhibition prevents GapDH2 from depleting the NADPH pool in such conditions and could explain the Δ*cp12* phenotypes in dark phases with supplied glucose. Inhibition of GapDH2 by Cp12 seems to be of higher relevance than binding of Prk for proper acclimation to glucose supply in dark phases (Lucius et al., 2022). However, we cannot rule out that Cp12 might have regulatory interactions with other proteins besides GapDH and Prk. In *Chlamydomonas reinhardtii*, Cp12 can bind aldolase as another interactor protein (Erales et al., 2008).

## 3 PirC contributes to the regulation of C/N homeostasis

Substantial amounts of organic carbon are used as precursors for N assimilation in cyanobacteria, which is basically done by the GS/GOGAT cycle. Because 2OG connects C and N metabolism in cyanobacteria, regulation of N assimilation under varying N supply is ultimately accompanied by regulations of carbon catabolism and anabolism (Forchhammer et al., 2022). Signal transduction via specific 2OG-binding proteins like PII and NtcA as well as NdhR triggers regulatory cascades for metabolic adjustment and maintenance of the C/N homeostasis. When combined N sources are limited, excess products of photosynthetic CO_2_ fixation are immediately redirected towards synthesis of glycogen (Klotz et al., 2016), while pigments are gradually degraded to refill soluble amino acid pools (e.g., Krauspe et al., 2021). Under prolonged N limitation, PHB slowly accumulates in granular structures promoted by glycogen turnover (Koch et al., 2019). The enhanced synthesis of glycogen reserves also serves as a preparation for recovery from chlorosis when nitrogen availability induces recovery of growth. The mobilization of stored glycogen during awakening from chlorosis supports the metabolism with energy and carbon skeletons (Doello et al., 2018). This is an essential process, as *Synechocystis* mutants deficient in glycogen synthesis do not enter chlorosis and die upon N starvation (Gründel et al., 2012).

### 3.1 Discovery of the PirC protein involved in C/N homeostasis

Many aspects related to the acclimation toward different C/N levels are regulated via the central regulatory protein PII. During the last years, an impressive number of PII-interacting proteins has been identified that play crucial roles in the nitrogen and carbon metabolism (Forchhammer et al., 2022). The latest one reported is PirC (“PII-interacting regulator of carbon metabolism”), a small protein (112 amino acids in *Synechocystis*, Sll0944) that is highly conserved among cyanobacteria (Orthwein et al., 2021). The *pirC* gene is located upstream of the gene for glycogen synthase GlgA1 required for glycogen synthesis that is highly activated during the acclimation to nitrogen deprivation. Initially, it had been identified as an interactor of unknown function with the protein PII in *Synechocystis* (Watzer et al., 2019). The gene and protein expression of PirC is highly responsive to different C/N ratios, upregulated and highly abundant in N starvation conditions (Spät et al., 2018), while repressed under LC conditions (Klähn et al., 2015; Spät et al., 2021; Barske et al., 2023). Recent co-immunoprecipitation studies identified Pgam1 (Slr1945) as second strong interaction partner with PirC in presence of 2OG and ATP.

As Pgam1 is a key enzyme of the triose-phosphate hub, this finding suggested PirC as an important regulatory link between central C and N metabolisms in *Synechocystis*. Previously, Pgam1 was predicted as a key control point in carbon metabolism from modelling data of *Synechococcus* 7942 (Jablonsky et al., 2014). Metabolic data from *Synechocystis* and *Synechococcus* 7942 cells shifted to different Ci suggest that under LC conditions, the Pgam1-catalysed export of 3PGA from the CBB cycle is increased to provide lower glycolysis and subsequent TCA cycle with newly fixed carbon, which sustains NH_4_^+^ assimilation via the GS/GOGAT cycle, diminishing glycogen synthesis. This is indicated by LC-shift induced accumulation of the Pgam1 reaction product 2PGA (Eisenhut et al., 2008b; Schwarz et al., 2011). Furthermore, the overexpression of PirC led to an increased glycogen accumulation in sufficient CO_2_ availability (Muro-Pastor et al., 2020) indicating a regulatory impact of PirC on carbon flux redirection.

**Figure 5:**
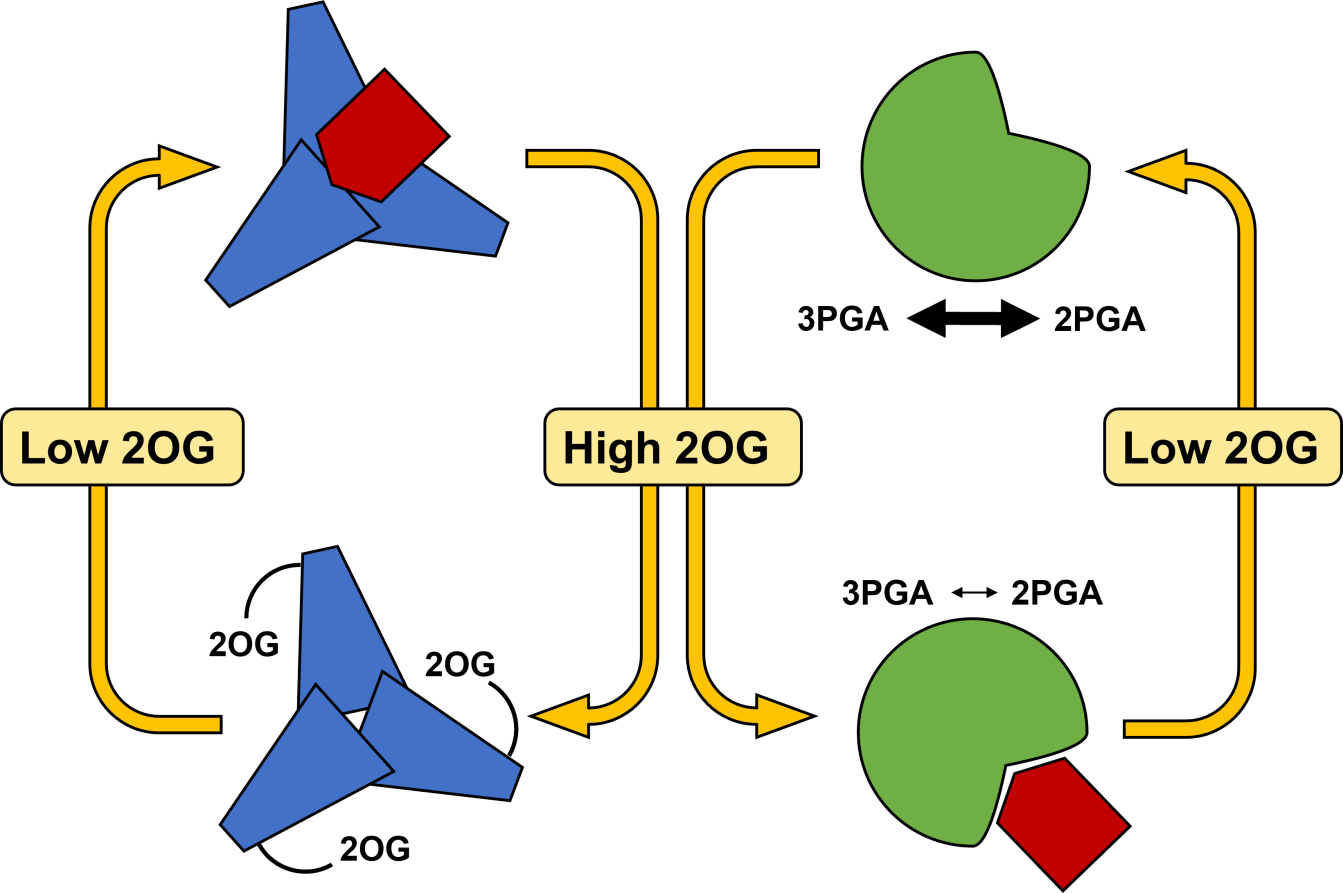
Model of regulation of central carbon metabolism under varying C/N ratios by PirC. The small PirC protein (red) can either be bound to trimeric PII (blue) under low 2OG levels or can be bound to PGAM (green) thereby decreasing the 3PGA/2PGA interconversion (small and thick arrows). The two states are regulated by varying 2-oxoglutarate levels signalling low C/N or high C/N ratios. 2OG, 2-Oxoglutarate; 2PGA, 2-Phosphoglycerate; 3PGA, 3-Phosphoglycerate; PII, trimeric PII protein (blue); PGAM, Phosphoglycerate mutase (green).

According to the current knowledge, the PII protein binds PirC at low 2OG levels, and releases it under high 2OG levels, which reflect N-sufficient and N-limiting conditions, respectively (**Figure 5**). Released PirC can then bind to Pgam1 and inhibits its activity at high cellular C/N ratios. The Pgam1 inhibition redirects carbon flux towards formation of glycogen storage. This facilitates adjustment of the C/N homeostasis and also prepares the cell for potential long-term N starvation (Orthwein et al., 2021). When cells are replenished with N sources, PirC is bound again to PII, and amounts of PirC decrease gradually (Doello et al., 2018). Muro-Pastor et al. (2020) showed that increased levels of PirC (alternatively named “CfrA”) result in a proportional glycogen accumulation. During resuscitation from nitrogen depletion, a PirC-deficient mutant displayed faster glycogen consumption and a more rapid re-greening compared to wild type. This indicated that in N resuscitation, a gradual degradation of PirC might be required to decrease a potentially inhibiting behaviour of this regulatory protein.

### 3.2 Impact of PirC on metabolome

The role of PirC regulation on a metabolic level was analysed in a N-shift experiment comparing short-term (48 h) N-depleted cells of wild type and Δ*pirC* (Orthwein et al., 2021). Similar to Ci shift experiments, such abrupt change of nutrient availability requires an immediate reaction to adjust metabolic flow and maintain the C/N homeostasis. Among the metabolic differences between both strains, the 2OG levels rose immediately in Δ*pirC* after the N starvation and remained elevated, while 2OG levels gradually decreased in wild type. This result shows that the absence of a functioning Pgam-PirC regulation causes a reduced metabolic flux towards the TCA cycle in N depletion, by which the initial responsive accumulation of 2OG is slowly wearing off. 3PGA levels remained low for Δ*pirC*, whereas increasing levels for the wild type indicate inhibition of Pgam by PirC. These observations confirm that in N depletion, regulation via PirC causes 3PGA accumulation that can be redirected to glycogen formation. Furthermore, rising 3PGA levels activate GlgC, which catalyses the conversion of G1P to ADP-glucose in the glycogen hub (Preiss and Romeo, 1994). This mechanism enables PirC-mediated stimulation of glycogen synthesis while lower glycolysis is simultaneously restricted. High pyruvate levels in Δ*pirC* indicate an increased conversion of 3PGA to 2PGA by flux through Pgam1, aligning with increased PHB levels derived from acetyl-CoA. Overall, the metabolic investigations deliver evidence that under N depletion, the Pgam downstream reaction is normally curbed by PirC as not enough N is available for subsequent amino acid biosynthesis.

So far, no fully segregated Pgam1 (Slr1945) mutant could be obtained for *Synechocystis*, preventing significant physiological studies on strains lacking this enzymatic function. However, these difficulties suggest that Pgam1 is essential for the central carbon metabolism of *Synechocystis*, and cannot be substituted by any other enzyme, thus enhancing the significance of Pgam1 as a key control point for carbon allocation. Equally, this finding makes the existence of other functional Pgam isoenzymes in *Synechocystis* unlikely, despite the annotation of at least one other potential Pgam in many cyanobacterial genomes.

The discovery of PirC as regulator for carbon allocation from the CBB cycle into lower glycolysis made it attractive to modify this regulatory step to direct organic carbon in the desired direction. The first biotechnological applications after characterization of PirC regulation was demonstrated to produce PHB (Koch et al., 2020). Under N starvation, there is a constant turnover of glycogen during progressing chlorosis and dormancy (Koch et al., 2019). This residual flux creates acetyl-CoA as precursor for PHB. In a PirC-deficient strain, this flux is increased because Pgam1 is uninhibited in low N conditions leading to a significant increase in PHB storage (Orthwein et al., 2021). The ability of Δ*pirC* strains to redirect carbon metabolism towards synthesis of PHB contributed to engineer a *Synechocystis* strain that showed the highest accumulation of PHB under nitrogen limitation so far, reaching over 80% PHB per cell dry weight (Koch et al., 2020). Beyond that, the PirC-deficient *Synechocystis* mutant was also used as chassis to increase ethanol titres. As expected from the higher flux of organic carbon into the pyruvate pool, a conditional increase of ethanol production was demonstrated in such engineered strains (Böhm et al., 2023). These findings clearly support the notion that a better understanding of regulatory aspects for carbon partitioning will have an important impact on using cyanobacteria as green cell factories.

Kinetic and binding studies revealed competitive inhibition of Pgam1 by PirC. In the future, a structural analysis of the complex formation could elucidate further details of the regulation. Other potential interaction partners of PirC have been identified by co-immunoprecipitation experiments. Among them are dihydrolipoamide dehydrogenase, dihydrolipoamide acetyltransferase, and pyruvate dehydrogenase E1 beta as three components of the pyruvate dehydrogenase (PDH) complex in *Synechocystis* (Muro-Pastor et al., 2020), as well as an ortholog of CcmP encoded by the gene *slr0169* (Orthwein et al., 2021). In *synechococcus 7942*, CcmP is a carboxysome shell protein with a central pore potentially functioning in metabolite transport, most likely the Pgam1 substrate 3PGA (Larsson et al., 2017). Identification of other regulatory properties of PirC for metabolite flux in the central carbon metabolism is therefore an interesting goal for future research.

## 4 Concluding remarks and outlook

Much progress has been made to broaden the knowledge about how cyanobacteria, particularly using the model strain *Synechocystis*, regulate their central carbon metabolism based on certain environmental conditions, like changing Ci availabilities as well as acclimation to different N levels. Several studies indicated that the enzyme Eda somehow contributes to glycogen breakdown during HC to LC shifts in *Synechocystis*. Future research on the postulated regulatory function of this protein will certainly increase the understanding of cyanobacterial glycogen metabolism during light phases. Other regulatory layers that determine the preference for a specific glycolytic route in dependence of certain environmental fluctuations could be targets for future biotechnological applications.

Further, recent results show that Cp12 contributes to redox regulation in conditions with supplied external glucose and can fine-tune the CBB cycle and carbohydrate metabolism in *Synechocystis*. This extends the role of Cp12 beyond dark-night regulation and also reveals contribution to acclimation to changing Ci conditions. If further potential targets or interactors of Cp12 can be identified, this could unravel an extended regulatory network of this versatile conditionally disordered protein. In this regard, another auspicious layer of regulation that requires further research efforts is the influence of phosphorylation of Cp12 itself and related effects on the CBB cycle.

Lastly, the novel PII-interactor PirC identifies phosphoglycerate mutase Pgam1 as a key control point of carbon storage metabolism in *Synechocystis*. The characterization of the PirC-mediated regulation of such a crucial branching point for carbon allocation harbours great potential for biotechnological applications as it implies new ways to redirect fluxes towards target metabolites or biopolymers such as PHB. Also, structural analysis of enzymatic complexes could help to further elucidate the inhibitory mechanism of PirC on Pgam1.

In conclusion, the cyanobacterial central metabolism is elusively complex where hidden regulatory layers and unknown protein interactions involved in the switch between photoautotrophy and heterotrophy will offer research topics for years to come.

## Author contributions

MH: Supervision, Writing – review & editing. SL: Writing – original draft.
